# Multi-objective optimization of wire electrical discharge machining process using multi-attribute decision making techniques and regression analysis

**DOI:** 10.1038/s41598-024-60825-w

**Published:** 2024-05-03

**Authors:** Masoud Seidi, Saeed Yaghoubi, Farshad Rabiei

**Affiliations:** 1 Department of Computer Engineering, Faculty of Engineering, Ilam University, Ilam, Iran; 2 Department of Mechanical Engineering, Faculty of Engineering, Ilam University, Ilam, Iran

**Keywords:** Electrical discharge machining, Roughness, Hardness, Dimensional accuracy, Method based on the removal effects of criteria, Weighted aggregates sum product assessment, Mechanical engineering, Mechanical properties

## Abstract

Wire electrical discharge machining (WEDM) is one of the most important non-traditional machining methods that is widely used in various industries. The present research work is concerned with the influences of process variables on quality of machined specimen obtained from WEDM process. The process parameters to manufacture mold structure included wire feed speed, wire tension and generator power, and in the current research, the effects of these variables on the aim factors, namely dimensional accuracy, hardness and roughness of product surface have been investigated, simultaneously. In order to obtain the optimal experiment, the multi-objective optimization with discrete solution area has been employed. Method based on the removal effects of criteria (MEREC) and weighted aggregates sum product assessment (WASPAS) techniques have been used with the aim of weighting the objective functions and discovering the best practical experiment. In the following, the regression analysis has been employed to study the effects of variables on response factors. A good correlation between the results gained from two analysis methods was observed. Based on MEREC-WASPAS hybrid technique, the weights of roughness, hardness and dimensional accuracy of machined part were calculated to about 89%, 9% and 2%, respectively. In the selected optimal experiment, the amount of wire feed speed, wire tension and generator power variables were considered to, in turn, 2 cm/s, 2.5 kg, and 10%.

## Introduction

In the manufacturing processes, it is important to have a mold with high precision and quality. This process was done with wood and stone in the past, but nowadays they use metal, plastic, polymers, elastomers, thermoplastic and thermosetting. In general, a mold is a tool for forming any kind of product in a way that helps the product (like a template) takes the desired form. One of the most important types of molds used in industries is a cutting mold. These shearing molds are one of the remarkable widely applied types of press dies, which are used to create cavities, holes, edges and grooves on the workpiece and include many different mechanisms. There are different methods to make the molds. Since the mold must have high dimensional accuracy, it is important to choose its manufacturing method. Electrical discharge machining (EDM) is a machining process in which a generator source is employed to generate a low-voltage and high-ampere spark for machining purposes^1^. One of the most accurate methods of making metal molds is using a wire-cut machine. Wire electrical discharge machining (WEDM) is a device that cuts parts through a thin wire that is inside distilled water or dielectric liquid^2^. By creating a spark between the part and the wire, this device causes momentary melting of that point, where the melting process takes place inside the same dielectric liquid. The material of the workpiece on which the cutting operation is performed can be aluminum^3^, copper^4^, brass, and steel^5^. The sparks produced in this device are completely visible when the water is clean, and the removal of chips can be seen with the naked eye^6^. In the WEDM operation, there is no contact between the wire and the workpiece, and the distance or gap that exists between them greatly increases the accuracy of the manufacturing process^7^. Due to the lack of contact between the wire and specimen, physical pressure similar to grinding and milling processes is not applied to the workpiece^8^. Therefore, this process eliminates mechanical stresses, noise and vibration during machining operation and can produce various materials with a thickness of 300 mm. For this reason, it can be said that wire-cut is a suitable option for making molds and industrial parts^9^ due to its high dimensional accuracy^10^ and good surface quality^11^. Proper dimensional accuracy leads to improvement of fatigue strength, wear resistance and corrosion resistance. With this regard, any effort to improve dimensional accuracy in this manufacturing process can be a significant step in the direction of producing precise and strong industrial parts^12^. Considering that the cutting dies produce a part in every cycle of a few seconds and are continuously subjected to cyclic loading, it is remarkable to investigate the fatigue phenomenon from this point of view. One of the factors that can increase the fatigue life of cutting molds is enhancing the hardness value^13^.

Several researches have been conducted to improve the quality of parts produced via WEDM process. Zahoor et al.^14^ optimized the dimensional accuracy and surface roughness of wire-cut process in Inconel 718 alloy using genetic optimization algorithm. Their results demonstrated that the dimensional accuracy depends on the clear time of the pulse, servo voltage and wire feeding. Chaudhary et al.^15^ studied on the effect of changing the input parameters on the optimization of dimensional accuracy in the manufacture of miniature gears by wire-cut operation. A mathematical model was derived using the Taguchi method. TOPSIS and ANOVA showed that the pulse light time is the most significant parameter for good dimensional accuracy. Paturi et al.^16^ focused on the influences of control factors such as pulse on time, pulse off time, peak current, voltage and wire feed rate on surface roughness of Inconel 718 steel manufactured part. Their results demonstrated that the peak current has 60.21% effect on the surface roughness. Nair et al.^17^ studied on the effect of changing different parameters on dimensional accuracy, material removal rate and surface roughness of Inconel 718 workpiece using wire-cut process. The outcomes gained from their research showed that the dimensional accuracy of machined holes decreases with increasing in wire tension. Arya and Singh^18^ optimized the parameters of pulse light time, pulse off time, peak current and server voltage, dimensional deviation and cutting rate in WEDM process. The results of the ANOVA in their research revealed that the pulse light time is the most influential parameter on the dimensional deviation. Ghasempour-Mouziraji et al.^19^ tried to minimize the geometrical deviation of parts produced via wire-cut using ANN and non-dominated sorting genetic algorithm (NSGA) methods. Wire speed, pulse time and feed rate were input variables in their study. This research work provided a new perspective to optimize the dimensional accuracy in the wire-cut process. Kiyak et al.^20^ investigated on the effect of pulse off time, pulse light time and mold thickness on steel hardness in WEDM operation. They reported the direct influences of these parameters on surface hardness. Ishfaq et al.^21^ focused on the optimization of EDM operation in order to reduce the geometric dimensional deviation and electrode wear. According to their findings, the performance of transformer oil in improvement of this process was the best choice in comparison with other dielectrics. An in-depth evaluation on the improvement of wire electrical discharge machining has been carried out by Chatterjee et al.^22^. They employ multi attribute decision making methods in intuitionistic fuzzy environment to achieve proper WEDM condition. Based on their findings, the optimal values of pulse-on time, pulse-off time, wire feed and wire tension were considered to, in turn, 115 μs, 55 μs, 3 m/min, and 7 kg-F. Tiwari et al.^23^ employed multi-criteria decision making (MCDM) model to optimize geometrical parameters in EDM process. In their research, the input variables include voltage, tool feed rate, and machining time, and the aim was to study the effects of aforementioned variables on radial overcut, circularity of the machined hole and heat-affected zone.

Based on the researches done, it can be said that checking the quality of the product obtained from the wire electrical discharge machining operation is of great importance. Various criteria have been introduced to investigate the quality of the produced sample, among which the hardness and roughness of the cut specimen surface can be mentioned. Several variables affect the quality of final product. Considering the importance of the three factors of roughness, hardness and dimensional accuracy of the manufactured part, in the current research, the effect of process variables on all three mentioned factors has been simultaneously investigated, which has not been performed in previous studies. The studied variables include wire feed speed, wire tension and generator power, and each of them is defined in three levels. In order to choose the best experimental test, method based on the removal effects of criteria and weighted aggregates sum product assessment technique has been used. Multi-objective optimization with discrete solution area is employed and using multi-attribute decision making techniques and regression analysis, the optimal condition is introduced. Multi-objective optimization with this approach has not been done so far.

## Experimental procedures

**Figure 1:**
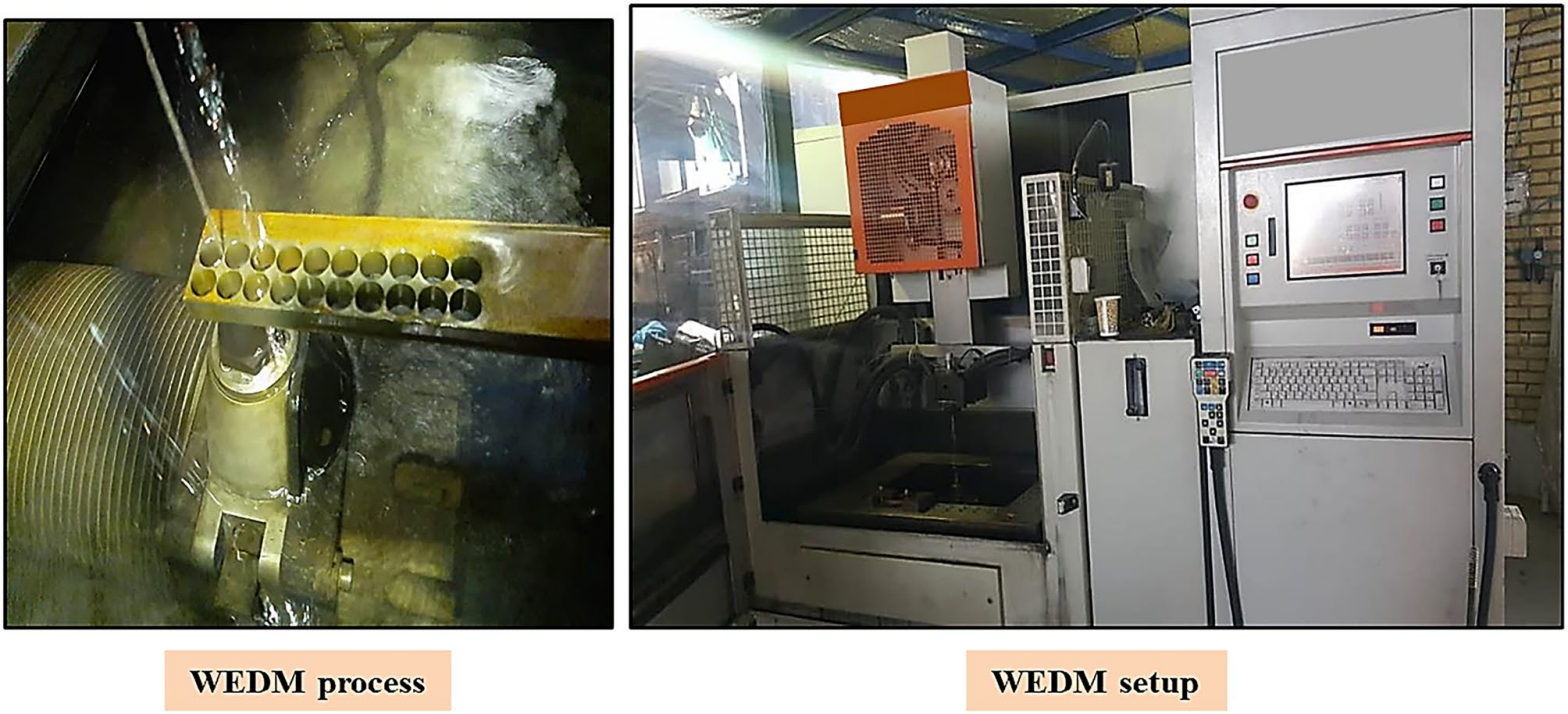
A view of experimental set used in current study.

One of the main steels used in manufacturing of molds is low Molybdenum alloy steel (Mo40), which is called St-4140 in AISI standard. This alloy is fragile due to the presence of chromium, and about 0.2% nickel is added to remove the brittleness. It should be noted that the resistance of these alloys is up to 500–600 °C. Carbon, Chromium, Manganese, Molybdenum and Silicon are alloy elements used in these steels. These materials have a high strength-to-weight ratio, because they are subjected to austenitizing, quenching, and then tempering operations. In the present research work, Mo40 steel was used as the workpiece material. Firstly, in order to achieve uniformity in thickness, the workpiece was completely machined using a shaper machine and brought to final dimensions of 320 mm × 40 mm and thickness of 10 mm. Then, it was fixed on the CHARMILLES 5-axis wire-cut machine. A view of experimental set is shown in Fig. 1. In the current study, wire feed speed, wire tension and generator power were selected as input variables. Based on previous researches and limitations of the setup, the values of pulse on time (TON), pulse off time (TOFF), current, and voltage have been considered to, in turn, 3 µs, 5 µs, 2 A, and 90 V. The wire material was copper with diameter of 0.2 mm and distilled water was used as the dielectric fluid. The ranges of these parameters are given in Table 1. It should be noted that the ranges of variables have been considered according to previous studies and setup constrains.

**Table 1:** The values of the input parameters in the present study.

Wire feed speed (cm/s)	Wire tension (kg)	Generator power (%)
2–6–10	0.5–1.5–2.5	10–30–50

The purpose of the present research work is to investigate the roughness, hardness and dimensional accuracy of the surface as a result of WEDM operations. In this study, R_a_, which shows the average deviation of the lows and highs from the middle line, has been chosen as a measure of surface roughness. In order to measure the surface roughness of the final product, Time 3110 portable roughness meter was employed. For each workpiece, the surface roughness test was measured 5 times with 8 mm length of probe, and after eliminating the outlier data, the average data was considered as surface roughness. Leeb Hardness Tester TA—with an accuracy of 0.1 has been used to calculate the surface hardness of the cut specimens. For manufactured parts, the hardness value was measured at four points and after removing the illogical data, the average data was considered as hardness of final product.

## Proposed solution approach

**Figure 2:**
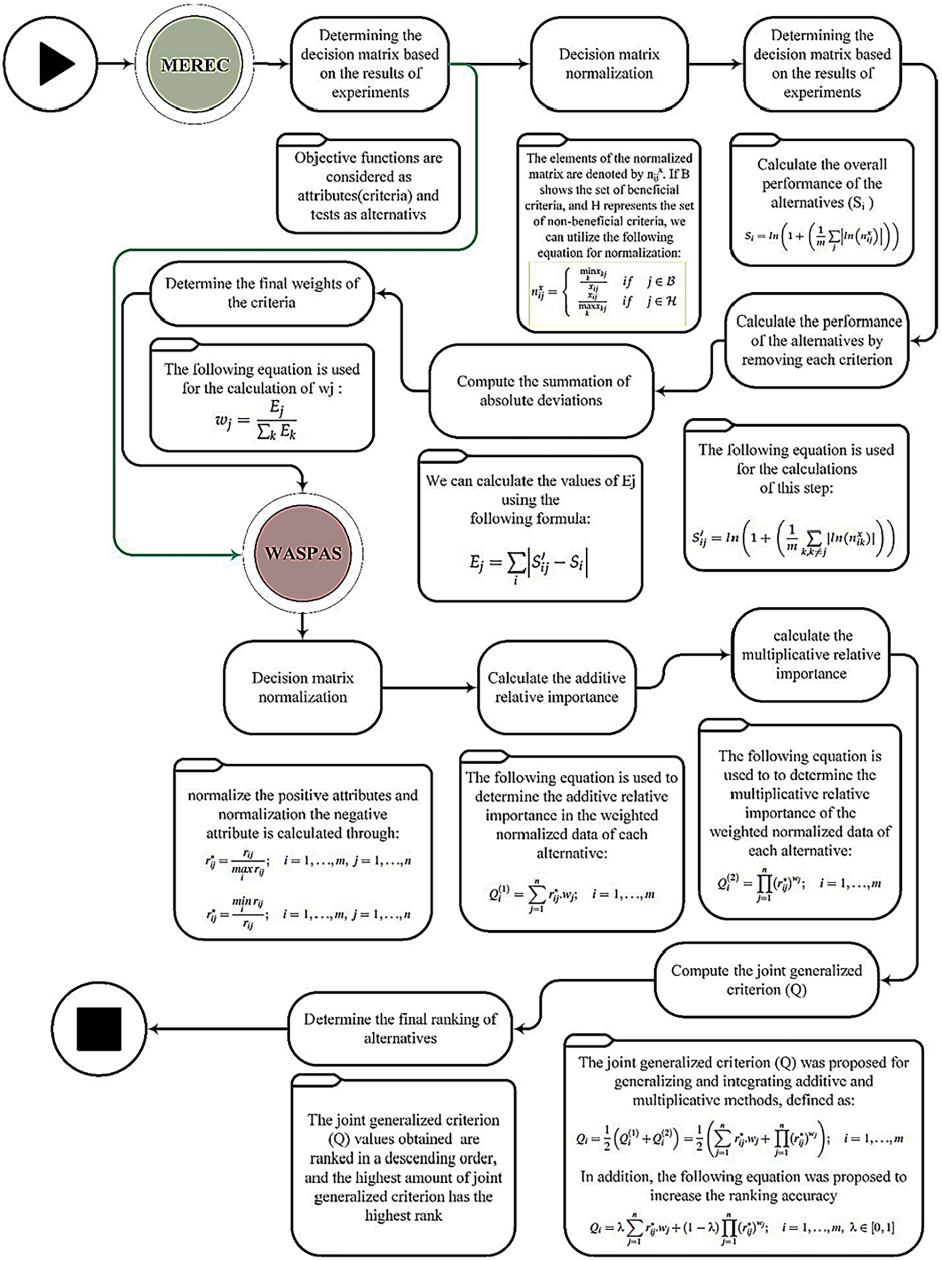
Proposed solution approach.

Optimization with discrete solution area is a category of optimization problems. Multi-attribute decision making (MADM) techniques are suitable and widely used tools for solving such problems. MADM techniques rank the alternatives employing attributes (criteria)^24^. Attributes are objective functions and alternatives are solutions. Using attributes and alternatives, a matrix is formed, which is called a decision matrix. The decision matrix is the most important input to MADM techniques. There are two categories of attributes: (1) Attribute with a positive aspect (an index whose higher value is more favorable) and (2) An attribute with a negative aspect (an index whose lower value is more favorable). The positive and negative attributes are objective functions whose goal is to maximize and minimize them, respectively. In this research, two techniques, namely WASPAS and MEREC are used. The weighted aggregates sum product assessment (WASPAS) method was introduced by Zavadskas et al.^25,26^. This method is a combination of weighted sum model (WSM) and weighted product model (WPM). WASPAS is one of the latest methods of choosing the best alternative in MADM techniques, which is obtained from the combination of previous methods. This method has more accuracy compared to independent ones. WASPAS has two inputs: the decision matrix and the weights of the attributes (criteria). At first, the attribute weights are obtained applying method based on the removal effects of criteria (MEREC) method. In the following, it is provided to WAPAS method as an input. MEREC is a weighting method which is introduced, recently. In multi-attribute decision making (MADM) techniques, before choosing the best alternative, the weight of criteria must be determined, which is often uses the opinions of experts. MEREC is the latest alternative-based method for determining the weight of criteria. The desirability of this method is due to the absence of errors corresponded to the subjective judgments of experts^27,28^. The used multi-attribute decision making techniques have a discrete solution space and select one alternative from among the available alternatives and are sensitive to the weight of criteria. All the steps related to determining the weight of criteria and ranking the alternatives with additional explanations are shown in Fig. 2.

## Results and discussions

In the present section, the findings of current research are presented. For this purpose, the results of experimental tests are given, firstly. In the following, in order to analyze the outcomes obtained from WEDM process, MEREC-WASPAS hybrid technique and regression analysis are implemented and the results of both methods are compared.

### Multi-attribute decision making approach

At first, the results of practical experiments obtained from wire electrical discharge machining operation are illustrated in this sub-section. In the following, the outcomes obtained from optimization with discrete solution area are illustrated. As explained earlier, in the current research, the aim is to optimize WEDM process variables. The input variables of wire feed speed, wire tension and generator power are considered in the design range given in Table 1.

**Figure 3:**
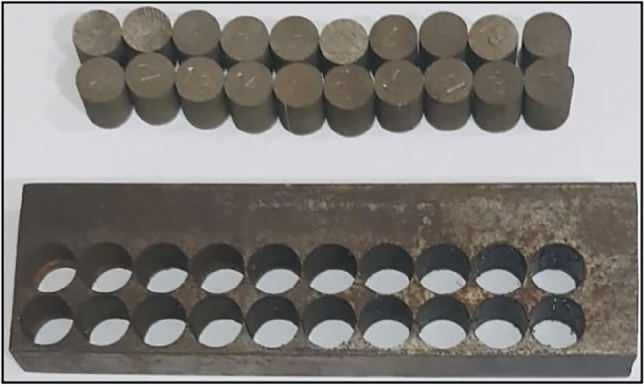
Produced specimens using wire-cut operation.

Considering that each variable has three levels, there will be 27 practical experiments in full factorial mode. In order to reduce the number of practical tests in wire-cut operation, the response surface methodology (RSM) was used and its number was decreased to 20 tests considering repeated ones. The experimental specimens obtained from the wire-cut operation are shown in Fig. 3. As it is known, for producing each product, a cylinder with a base diameter of 10 mm and a height of 10 mm is cut from the workpiece. The objective functions include dimensional accuracy, hardness, and roughness of product surface, and their values have been calculated according to the practical test.

Table 2 shows the results of practical tests. In the following, the normalized decision matrix using the method mentioned in Fig. 2 is given. This matrix is the input to the MEREC method (Eq. 1). Calculations related to the MEREC method are given in Table 3. The steps of MEREC method are shown in Fig. 2. As can be seen, the weight of dimensional accuracy, hardness and roughness of machined part are computed to 0.01693, 0.08901 and 0.89406, respectively. The maximum weight is related to surface roughness attribute. Then, the normalized decision matrix using the method mentioned in Fig. 2 is given. This matrix is the input to the WASPAS technique (Eq. 2). The outcomes obtained from WASPAS method are given in Tables 4 and 5. It should be mentioned that the steps of WASPAS method are shown in detail, previously (Fig. 2). After implementing WASPAS method, test No. 2 was chosen as the best alternative. According to this, the optimal values of the input variables consisting of wire tension, wire feed speed, and generator power were considered to 2.5 kg, 2 cm/s, and 10%, respectively. The second and third ranks are also highlighted in Tables 4 and 5. As it is clear, by changing the value of λ-parameter in WASPAS technique, the ranking of options does not change. If the λ-value equals to 0 and 1, WASPAS method is converted into the WSM and WPM techniques, respectively. Therefore, it can be concluded that the ranking obtained from WASPAS, WSM and WPM is the same.

1 $$ D\,\,(MEREC) = \left\{ {\begin{array}{*{20}{c}} {0.994}&{0.953}&{0.687} \\ {0.995}&{0.938}&{0.536} \\ {0.994}&{0.980}&{0.758} \\ {0.995}&{1.000}&{0.726} \\ {0.994}&{0.938}&{0.906} \\ {0.993}&{0.980}&{0.762} \\ {0.933}&{1.000}&{0.864} \\ {0.992}&{0.945}&{1.000} \\ {0.993}&{0.980}&{0.838} \\ {0.993}&{1.000}&{0.615} \\ {0.992}&{0.945}&{0.809} \\ {0.993}&{0.968}&{0.666} \\ {0.993}&{1.000}&{0.551} \\ {0.994}&{0.945}&{0.615} \\ {0.993}&{0.968}&{0.821} \\ {0.992}&{0.982}&{0.906} \\ {1.000}&{0.945}&{0.817} \\ {0.994}&{0.968}&{0.674} \\ {0.993}&{0.982}&{0.664} \\ {0.995}&{0.951}&{0.774} \end{array}} \right\}  $$

2 $$ D\;(WASPAS) = \left\{ {\begin{array}{*{20}{c}} {0.9978}&{0.9840}&{0.7802} \\ {0.9070}&{1.0000}&{1.0000} \\ {0.9981}&{0.9564}&{0.7064} \\ {0.9969}&{0.9376}&{0.7376} \\ {0.9980}&{1.0000}&{0.5916} \\ {0.9988}&{0.9564}&{0.7029} \\ {0.9987}&{0.9376}&{0.6200} \\ {0.9996}&{0.9922}&{0.5358} \\ {0.9989}&{0.9564}&{0.6396} \\ {0.9992}&{0.9376}&{0.8711} \\ {0.9998}&{0.9922}&{0.6620} \\ {0.9992}&{0.9684}&{0.8045} \\ {0.9988}&{0.9376}&{0.9726} \\ {0.9979}&{0.9922}&{0.8711} \\ {0.9988}&{0.9684}&{0.6528} \\ {1.0000}&{0.6550}&{0.5916} \\ {0.9919}&{0.9922}&{0.6558} \\ {0.9977}&{0.9684}&{0.7955} \\ {0.9988}&{0.9550}&{0.8068} \\ {0.9968}&{0.9854}&{0.6926} \end{array}} \right\} $$

**Table 2:** Decision matrix obtained from practical tests.

Aspect			+	+	−
Alternative					
Tension	Speed	Power	Dimensional accuracy (mm)	Hardness (Rockwell B- scale)	Surface roughness (µm)
0.5	2	50	9.973	69.450	3.64
2.5	2	10	9.965	70.575	2.84
2.5	6	30	9.976	67.500	4.02
0.5	10	10	9.964	66.175	3.85
0.5	6	30	9.975	70.575	4.80
1.5	6	30	9.983	67.500	4.04
1.5	6	50	9.982	66.175	4.58
1.5	6	30	9.991	70.025	5.30
1.5	6	30	9.984	67.500	4.44
1.5	6	30	9.987	66.175	3.26
2.5	10	50	9.993	70.025	4.29
1.5	10	30	9.987	68.350	3.53
2.5	10	10	9.983	66.175	2.92
1.5	6	10	9.974	70.025	3.26
1.5	6	30	9.983	68.350	4.35
1.5	2	30	9.994	67.400	4.80
2.5	2	50	9.914	70.025	4.33
0.5	10	50	9.972	68.350	3.57
1.5	6	30	9.983	67.400	3.52
0.5	2	10	9.981	71.025	3.02
Max			9.994	70.575	5.30
Min			9.914	66.175	2.84

**Table 3:** Calculation's worksheet gained from MEREC method.

${S}_{i}$	${S}_{i1}{\prime}$	${S}_{i2}{\prime}$	${S}_{i3}{\prime}$	$\left|{S}_{i1}{\prime}-{S}_{i}\right|$	$\left|{S}_{i2}{\prime}-{S}_{i}\right|$	$\left|{S}_{i3}{\prime}-{S}_{i}\right|$
0.44401	0.43905	0.4029	0.06813	0.00496	0.04111	0.37588
0.64261	0.63909	0.5976	0.0865	0.00351	0.04501	0.5561
0.33162	0.32579	0.31295	0.03332	0.00583	0.01867	0.29831
0.35211	0.3475	0.35211	0.00652	0.00461	0	0.34559
0.19924	0.19269	0.12821	0.0877	0.00656	0.07103	0.11154
0.32764	0.32112	0.30889	0.0342	0.00652	0.01875	0.29344
0.18123	0.17379	0.18123	0.00885	0.00744	0	0.17239
0.08026	0.07094	0.01001	0.08026	0.00933	0.07026	0
0.23514	0.22788	0.21456	0.03432	0.00726	0.02058	0.20082
0.4955	0.48967	0.4955	0.00949	0.00583	0	0.486
0.30651	0.29889	0.25089	0.0805	0.00762	0.05563	0.22601
0.45737	0.45131	0.4304	0.05029	0.00605	0.02697	0.40708
0.57884	0.57378	0.57884	0.00898	0.00507	0	0.56987
0.53832	0.53373	0.49446	0.07822	0.00459	0.04386	0.46011
0.26838	0.26146	0.23571	0.0498	0.00692	0.03267	0.21859
0.1511	0.14208	0.13038	0.03372	0.00902	0.02071	0.11738
0.2899	0.2899	0.23332	0.07094	0	0.05658	0.21896
0.4468	0.44194	0.41954	0.04843	0.00486	0.02726	0.39837
0.44781	0.44203	0.43245	0.03233	0.00578	0.01535	0.41548
0.3399	0.33533	0.29278	0.06866	0.00457	0.04713	0.27124
${E}_{j}$	0.11633		0.61158		6.14315	
${W}_{j}$	0.01693		0.08901		0.89406

**Table 4:**
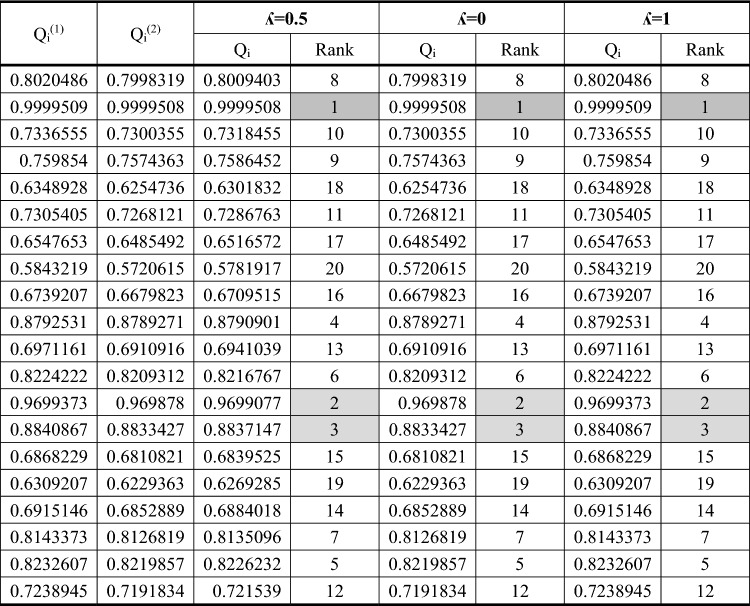
Calculation's worksheet obtained from WASPAS method for λ = 0, 0.5, and 1.

**Table 5:**
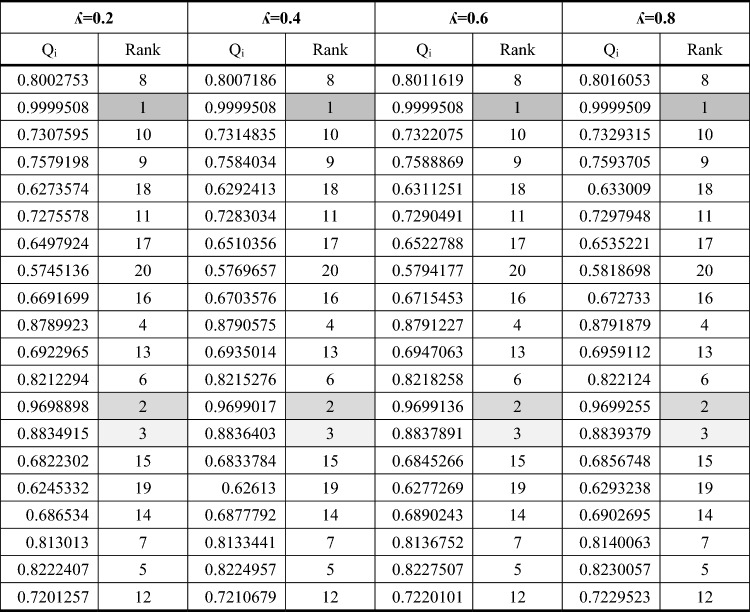
Calculation's worksheet obtained from WASPAS method for λ = 0.2, 0.4, 0.6, and 0.8.

Wire feed speed and wire tension are both parameters of wire movement control. The wire feeding speed indicates the length of the wire that is unrolled from the reel in a unit of time. Unnecessary increase of this parameter leads to growth in wire consumption. On the other hand, being too low will lead to an increase in the possibility of the wire breaking. Considering the irregularities in the wire movement path, choosing the appropriate value for this parameter is most effective in reducing wire vibrations. Wire tension is a longitudinal force that is applied to the wire by machine to keep it in a straight line. Due to the presence of processing forces and the flexibility of the wire, there is always the possibility of the wire bending, which can be reduced by controlling this variable. If proper tension is applied on the wire, the deviation from the path, vibration and deformation will be effectively reduced and as a result, the machining error will be decreased. To create stable machining accuracy, a certain amount of tension in the wire is required to limit deviation of the path, bending and vibration. An increase in wire tension (in considerable ranges) can enhance the dimensional accuracy of final product, significantly. During the sparking, the wire moves at a nearly uniform speed over the workpiece. Due to the impact force of the spark on the wire, it is expected that the wire will vibrate under this impact. By increasing the amount of impact, the wire vibrates with a higher frequency. For this reason, it can be concluded that the roughness of the cut surface will decrease by enhancing the tension of the wire in this operation. With increasing generator power, the energy of sparks enhances, as a result, the volume of craters created on the surface of the workpiece increases. In this situation, the temperature on the surface of the workpiece is very high and due to these major sparks, bigger holes are created due to evaporation and melting of materials. For this reason, it can be said that increasing the generator power increases the cutting speed and decreases the smoothness of the surface in final product. Based on the explanations given above, it can be concluded that the outcomes obtained from practical experiments and multi-objective optimization seem reasonable.

### Regression analysis

**Figure 4:**
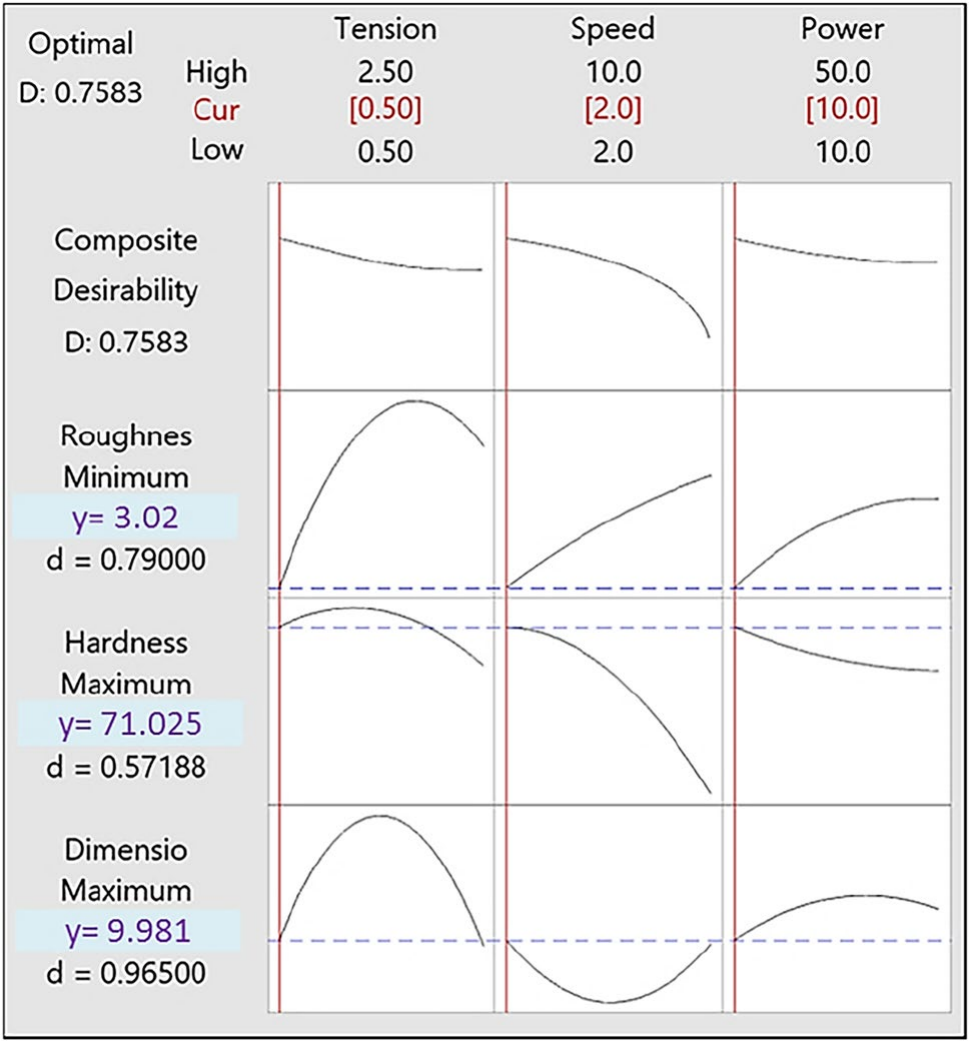
Response optimization curves for three objective functions.

**Figure 5:**
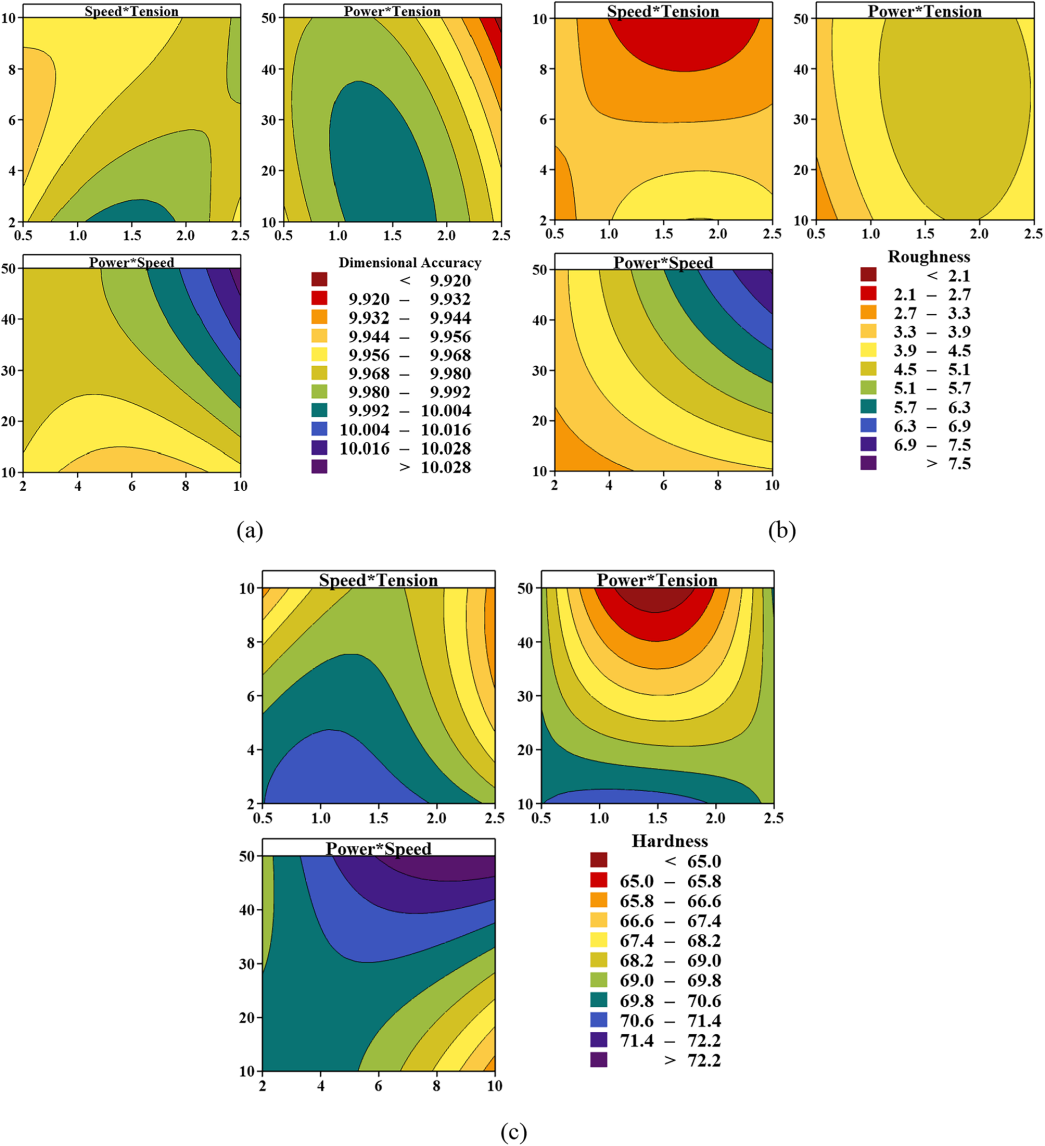
Contour plots presented for objective functions, namely (**a**) dimensional accuracy, (**b**) hardness, and (**c**) surface roughness.

In the present sub-section, the relationships between three objective functions and three input factors have been extracted via regression analysis. Using Minitab software, these relationships have been derived for three response variables as follows (Eqs. 3–5). According to the regression equations and without considering the weight for the response variables, the optimal solution has been obtained using Minitab software (Fig. 4). As can be seen, the best answer is gained by the Tension = 0.5 kg, Speed = 2 cm/s and Power = 10%. For these values, the amount of response factors, namely dimensional accuracy, hardness, surface roughness were computed to, in turn, 9.981 mm, 71.025 Rockwell B, and 3.020 μm. Based on these findings, an index called desirability has been calculated for the answer. The closer the value of desirability is to one, the more suitable the answer is. The desirability in the answer is obtained to 0.7583. According to regression equations, the contour plots for the three response variables are presented in Fig. 5.

3 $$\text{Dimensional Accuracy}=9.9259 + 0.1282 \times \text{Tension }- 0.01172 \times \text{Speed }+ 0.001294 \times {\text{Power}}- 0.0462\times {{\text{Tension}}}^{2} + 0.001357\times {{\text{Speed}}}^{2}- 0.000018\times {{\text{Power}}}^{2}- 0.00801\times \text{ Tension}*\text{Speed }- 0.000840\times \text{ Tension}\times {\text{Power}}+ 0.000197\times \text{ Speed}\times {\text{Power}}+ 0.00585\times {{\text{Tension}}}^{2}\times {\text{Speed}}+ 0.000046\times {{\text{Tension}}}^{2}\times {\text{Power}}- 0.000682\times \text{ Tension}\times {{\text{Speed}}}^{2}- 0.000005\times \text{ Tension}\times {\text{Speed}}\times {\text{Power}}$$

4 $${\text{Hardness}}=68.72 + 6.5 \times \text{Tension }+ 0.04 \times \text{Speed }+ 0.062\times \text{ Power }- 2.02 \times {{\text{Tension}}}^{2}- 0.102 \times {{\text{Speed}}}^{2}+ 0.00069\times {{\text{Power}}}^{2}+ 0.05\times \text{ Tension}\times {\text{Speed}}- 0.441\times \text{ Tension}\times \text{Power }+ 0.0281\times \text{ Speed}\times \text{Power }- 0.253\times {{\text{Tension}}}^{2}\times {\text{Speed}}+ 0.158\times {{\text{Tension}}}^{2}\times \text{Power }+ 0.070\times \text{ Tension}\times {{\text{Speed}}}^{2}- 0.0070\times \text{ Tension}\times {\text{Speed}}\times {\text{Power}}$$

5 $${\text{Roughness}}=-0.30 + 5.69\times \text{ Tension }+ 0.55 \times \text{Speed }+ 0.0438 \times \text{Power }- 1.55 \times {{\text{Tension}}}^{2}- 0.009\times {{\text{Speed}}}^{2} - 0.00058\times {\text{ Power}}^{2}- 1.015\times \text{ Tension}\times {\text{Speed}}- 0.017\times \text{ Tension}\times \text{Power }+ 0.0105\times \text{ Speed}\times \text{Power }+ 0.275\times {{\text{Tension}}}^{2}\times {\text{Speed}}+ 0.0052\times {{\text{Tension}}}^{2}\times \text{Power }+ 0.0068\times \text{ Tension}\times {{\text{Speed}}}^{2}- 0.0028\times \text{ Tension}\times {\text{Speed}}\times {\text{Power}}$$

In this section, the aim is to compare the outcomes obtained from MEREC-WASPAS hybrid technique and regression analysis. Based on the results extracted from both analysis methods, it was observed that the optimal values of the wire feed speed and generator power variables are the same for both analysis methods and the only difference is in the optimal value wire tension variable. As mentioned earlier, the amount of wire tension has a significant effect on surface roughness and dimensional accuracy of the mold produced via WEDM process. Increasing this variable has a significant effect on improving surface quality and dimensional accuracy. Since in the MEREC-WASPAS technique, the weight of the surface roughness factor was much higher than the other two factors, the selection of optimal variables was considered based on the maximum reduction in this response factor. Considering that in the regression analysis, the weight of all objective functions is considered the same, therefore, the maximum value of the wire tension variable (2.5 kg) was obtained for it in order to increase the maximum dimensional accuracy and surface quality, simultaneously. Accordingly, the findings obtained from the regression analysis also seem reasonable.

## Conclusions

In the present study, the effect of manufactured part using wire electrical discharge machining operations on the quality of the obtained products has been investigated, experimentally. The material used in this research is Mo40 steel. The considered variables include wire feed speed, wire tension and generator power, and the purpose of the study is to investigate the effects of these variables on the three factors of hardness, roughness and dimensional accuracy of the product surface manufactured by WEDM operation. In order to perform multi-objective optimization and select the best practical test, two techniques, called method based on the removal effects of criteria (MEREC) and weighted aggregates sum product assessment (WASPAS) have been used. In the following, the regression analysis was investigated by considering the same weight for the objective functions. A summary of the results obtained is given below:

- Based on MEREC technique, the weight of objective functions consisting of dimensional accuracy, hardness and roughness of product surface were calculated to 0.01693, 0.08901 and 0.89406, respectively.
- According to WASPAS technique, test No. 2 was selected as the best choice. In this state, the optimal amounts of wire tension, wire feed speed, and generator power were considered to 2.5 kg, 2 cm/s, and 10%, respectively.
- By changing the λ-value in WASPAS technique, the ranking of the superior practical tests did not change, which indicated the robustness of the optimization with discrete solution area in this case study.
- In choosing the optimal test, the difference between the two methods of MEREC-WASPAS and regression analysis was related to wire tension variable. Due to the fact that the weight coefficients of the target factors are the same in this method, its maximum value (2.5 kg) was considered as the best experimental test.

## Data Availability

The datasets generated and analyzed during the current study are available from the corresponding author on reasonable request.

## Abbreviations

ANN: Artificial neural network
ANOVA: Analysis of variance
EDM: Electrical discharge machining
MADM: Multi-attribute decision making
MCDM: Multi-criteria decision making
MEREC: Method based on the removal effects of criteria
NSGA: Non-dominated sorting genetic algorithm
RSM: Response surface methodology
TOPSIS: Technique for order of preference by similarity to ideal solution
WASPAS: Weighted aggregates sum product assessment
WEDM: Wire electrical discharge machining
WPM: Weighted product model
WSM: Weighted sum model
